# Outcome Prediction of Postanoxic Coma: A Comparison of Automated Electroencephalography Analysis Methods

**DOI:** 10.1007/s12028-022-01449-8

**Published:** 2022-03-02

**Authors:** Stanley D. T. Pham, Hanneke M. Keijzer, Barry J. Ruijter, Antje A. Seeber, Erik Scholten, Gea Drost, Walter M. van den Bergh, Francois H. M. Kornips, Norbert A. Foudraine, Albertus Beishuizen, Michiel J. Blans, Jeannette Hofmeijer, Michel J. A. M. van Putten, Marleen C. Tjepkema-Cloostermans

**Affiliations:** 1 Department of Neurology, Rijnstate Hospital, Arnhem, The Netherlands; 2 Department of Clinical Neurophysiology, Technical Medical Centre, University of Twente, Enschede, The Netherlands; 3 Department of Neurology, Donders Institute for Brain, Cognition, and Behaviour, Radboud University Medical Centre, Nijmegen, The Netherlands; 4 Department of Clinical Neurophysiology, St. Antonius Hospital, Nieuwegein, The Netherlands; 5 Department of Intensive Care, St. Antonius Hospital, Nieuwegein, The Netherlands; 6 Department of Neurology and Neurosurgery, University Medical Center Groningen, University of Groningen, Groningen, The Netherlands; 7 Department of Critical Care, University Medical Center Groningen, University of Groningen, Groningen, The Netherlands; 8 Department of Neurology, VieCuri Medical Center, Venlo, The Netherlands; 9 Department of Intensive Care, VieCuri Medical Center, Venlo, The Netherlands; 10 Intensive Care Center, Medisch Spectrum Twente, Enschede, The Netherlands; 11 Department of Intensive Care, Rijnstate Hospital, Arnhem, The Netherlands; 12 Department of Clinical Neurophysiology and Neurology, Medisch Spectrum Twente, P.O. 50000, 7500 KA Enschede, The Netherlands

**Keywords:** Brain hypoxia, Cardiac arrest, Deep neural networks, Electroencephalography, Machine learning, Prognosis

## Abstract

**Background:**

To compare three computer-assisted quantitative electroencephalography (EEG) prediction models for the outcome prediction of comatose patients after cardiac arrest regarding predictive performance and robustness to artifacts.

**Methods:**

A total of 871 continuous EEGs recorded up to 3 days after cardiac arrest in intensive care units of five teaching hospitals in the Netherlands were retrospectively analyzed. Outcome at 6 months was dichotomized as “good” (Cerebral Performance Category 1–2) or “poor” (Cerebral Performance Category 3–5). Three prediction models were implemented: a logistic regression model using two quantitative features, a random forest model with nine features, and a deep learning model based on a convolutional neural network. Data from two centers were used for training and fivefold cross-validation (*n* = 663), and data from three other centers were used for external validation (*n* = 208). Model output was the probability of good outcome. Predictive performances were evaluated by using receiver operating characteristic analysis and the calculation of predictive values. Robustness to artifacts was evaluated by using an artifact rejection algorithm, manually added noise, and randomly flattened channels in the EEG.

**Results:**

The deep learning network showed the best overall predictive performance. On the external test set, poor outcome could be predicted by the deep learning network at 24 h with a sensitivity of 54% (95% confidence interval [CI] 44–64%) at a false positive rate (FPR) of 0% (95% CI 0–2%), significantly higher than the logistic regression (sensitivity 33%, FPR 0%) and random forest models (sensitivity 13%, FPR, 0%) (*p* < 0.05). Good outcome at 12 h could be predicted by the deep learning network with a sensitivity of 78% (95% CI 52–100%) at a FPR of 12% (95% CI 0–24%) and by the logistic regression model with a sensitivity of 83% (95% CI 83–83%) at a FPR of 3% (95% CI 3–3%), both significantly higher than the random forest model (sensitivity 1%, FPR 0%) (*p* < 0.05). The results of the deep learning network were the least affected by the presence of artifacts, added white noise, and flat EEG channels.

**Conclusions:**

A deep learning model outperformed logistic regression and random forest models for reliable, robust, EEG-based outcome prediction of comatose patients after cardiac arrest.

## Introduction

Approximately half of all comatose patients admitted after cardiac arrest at the intensive care unit (ICU) never regain consciousness [1]. Neurological outcome prediction can facilitate communication between doctors and relatives and prevent futile treatment in case of poor outcome perspectives. Bilateral absence of somatosensory evoked potentials (SSEPs) is a widespread, reliable predictor of poor outcome but has a relatively low sensitivity [2–4]. Electroencephalography (EEG) can add to reliable prediction of good or poor neurological outcome [5–11]. Generalized suppression or synchronous patterns with at least 50% suppression between 6 h and 5 days after cardiac arrest have been invariably associated with poor outcome [12]. A continuous background pattern at 6 or 12 h was an independent predictor of good outcome [13–15].

Visual analysis of the EEG is the current gold standard and is included in guidelines and clinical practices [16, 17]. Visual EEG analysis yields reliable outcome prediction in approximately half of all patients [6, 10, 12, 18]. An important drawback of visual EEG analysis is the inability to capture the integral richness of the EEG signal. Furthermore, visual analysis can only be performed by experienced electroencephalographers, is time consuming, and is subject to intraobserver and interobserver variability [19, 20]. Automated EEG analysis may overcome these limitations and can be performed at the bedside in real time in the ICU [20–24].

In previous work, we have introduced the cerebral recovery index (CRI), representing a probability of good outcome after cardiac arrest on the basis of the EEG that can be extracted at the bedside [20, 21]. The original CRI allowed reliable prediction of neurological outcome using five quantitative EEG features, including continuity, amplitude, and frequency content [21]. We significantly improved the CRI by adding more quantitative features and using a random forest model [20]. In more recent work, we used a logistic regression model in a new prediction algorithm on the basis of only two prespecified EEG features reflecting continuity and amplitude ratio [22]. In our latest work in this field, we used deep learning of a convolutional neural network (CNN), avoiding the need for explicit feature definition and using the ability to learn from the data. With all approaches, we achieved a similar or even better prognostic performance than with visual analysis [24].

The direct comparison of performance of these three prediction models on the same datasets has not been performed, and model robustness to artifacts has not been evaluated. In this study, we perform a head-to-head comparison between logistic regression, random forest, and deep learning models for EEG-based prediction of neurological outcome of patients with postanoxic coma. We train and evaluate the models on the same dataset and perform a comparative analysis of prognostic performance and robustness to artifacts in an external validation set.

## Methods

### Study Design and Participants

This is a retrospective analysis of data acquired in multiple prospective cohort studies. All studies included consecutive adult comatose (Glasgow Coma Scale ≤ 8) patients after cardiac arrest admitted to the ICUs of five teaching hospitals in the Netherlands (Medisch Spectrum Twente, Rijnstate Hospital, St. Antonius Hospital, University Medical Center Groningen, and VieCuri Medical Center). Parts of the data have been used in previous publications [12, 18, 20–24]. The Medical Ethical Committee Twente approved the protocol and waived the need for informed consent officially in 2019 (K19-11) because the EEG data were anonymized and EEG monitoring and clinical follow-up are part of current care in the participating centers.

### Standard of Care and Treatment Withdrawal

Patients were treated according to standard protocols, including targeted temperature management at 33 °C or 36 °C. Propofol, midazolam, and/or sevoflurane were used for sedation, and morphine, fentanyl, or remifentanil was used for analgesia. The decision of the withdrawal of life supporting treatment was considered only during normothermia, off sedation, and later than 72 h after cardiac arrest and were based on international guidelines (bilateral absence of SSEPs, absent or extensor motor responses, and absence of brainstem reflexes). Decisions on treatment withdrawal were sporadically taken between 48 and 72 h in cases of absent brainstem reflexes or SSEP responses. The EEG recorded in the first 72 h after cardiac arrest was not taken into account in decision making on treatment withdrawal.

### Outcome

The primary outcome measure of this study was neurological functional recovery 6 months after cardiac arrest expressed as a score on the Cerebral Performance Category (CPC) scale. Outcome was dichotomized as good (CPC 1–2, no or mild neurological impairment) or poor (CPC 3–5, severe neurological impairment, vegetative state, or death). Outcome was assessed at 6 months during a standardized telephone interview based on a Dutch translation of the EuroQol-6D questionnaire, except for one center in which CPC scores were assessed by using the Short Form 36 questionnaire.

### Continuous EEG Recordings and Preprocessing

Continuous EEG recording was started as early as possible after ICU admission until the patient regained consciousness, died, or up to 3–5 days otherwise. EEGs were recorded with 21 silver/silver chloride cup electrodes placed on the scalp according to the international 10–20 system. A computer algorithm, as applied in previous quantitative EEG studies [21], was used to select 5-min epochs with the least number of artifacts at every hour, 4–72 h after cardiac arrest. If all epochs at a time point after cardiac arrest contained too many artifacts, the respective time point was skipped. EEGs were transformed to a longitudinal bipolar montage. Subsequently, an artifact rejection algorithm, introduced in previous work [22], rejected EEG channels in all selected epochs with muscle activity or high amplitudes, and flat channels. Channels were excluded if the threshold of at least one of the artifact types was crossed. All threshold values were based on previous work [22]. For the CNN, a fixed input size was required and channels containing artifacts were not removed to maintain a consistent input for the networks, representing real-life EEG analysis. If more than six EEG channels contained artifacts, the entire epoch was excluded from further analysis for all models. Following artifact rejection, a sixth order bandpass Butterworth filter was applied (bandwidth 0.3–25 Hz). The 5-min EEG epochs were split into 30 segments of 10 s and entered into the models.

### Quantitative EEG Prediction Models

**Fig. 1:**
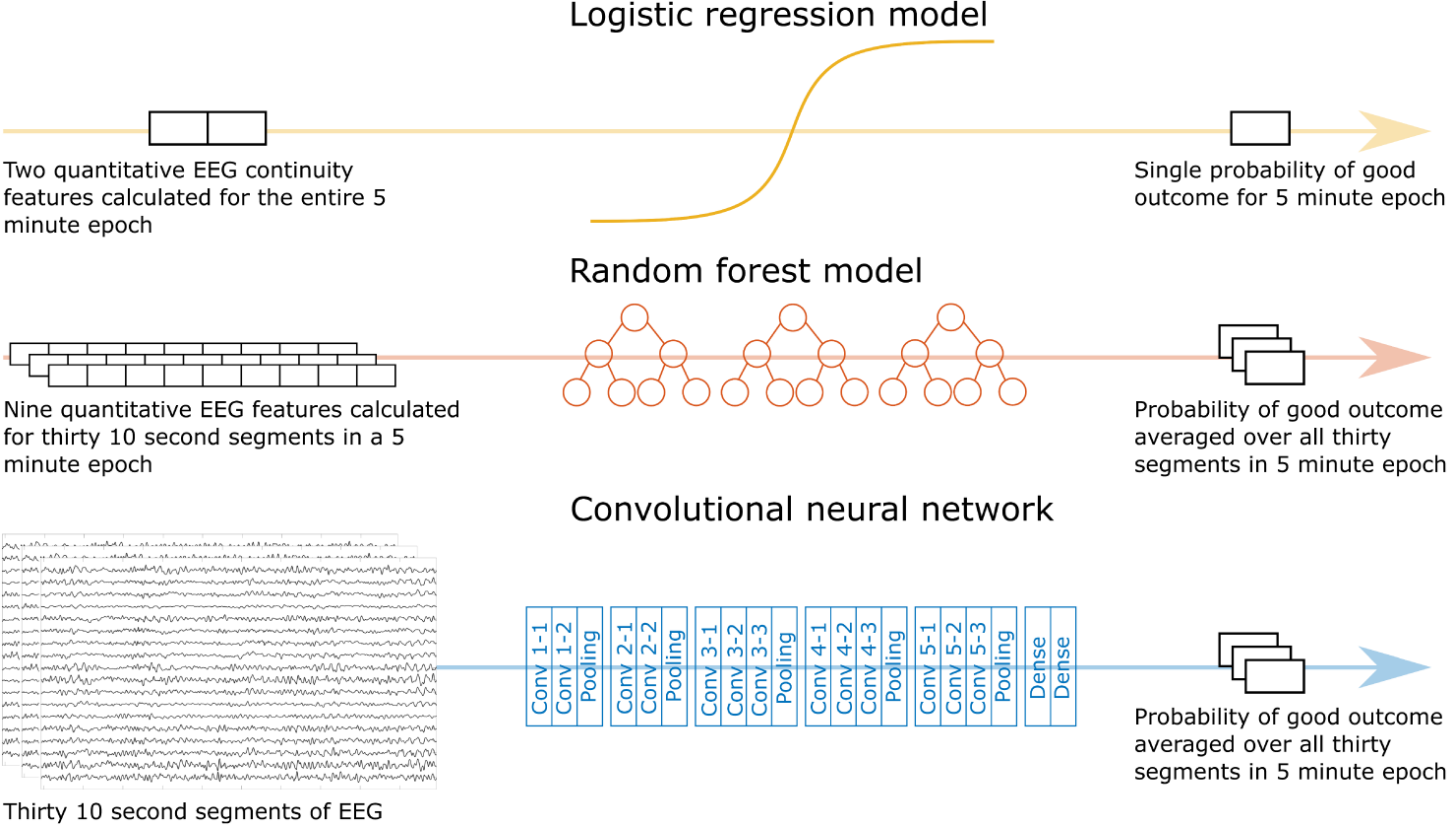
An overview of the automatic electroencephalogram (EEG) prediction models used in this study. The inputs of the respective models are shown on the left side and the outputs are shown on the right. The logistic regression model used two quantitative features as input and outputted the probability of good outcome by using a regression model with constants optimized during training. The random forest model used nine quantitative features for 30 10-s EEG segments as inputs and operated by using an ensemble of random independent decision trees to generate a probability of good outcome for all 30 segments. The convolutional neural network used raw 10-s segments of EEG as the input and performed feature extraction and classification by using convolutional filters. The output of the neural network was the probability of good outcome for all 30 segments

The logistic regression model used two predefined quantitative EEG continuity features computed for the entire 5-min epoch as input and generated a single probability for good outcome of the epoch [22]. The random forest model, based on 500 individual decision trees with five terminal nodes, took nine predefined, automatically calculated, quantitative EEG features as input and computed a probability of good outcome as an output for every 10-s segment [20]. Feature extraction was performed in MATLAB (MATLAB release R2019b, MathWorks Inc.). The CNN, based on a VGG model C network by the Oxford Visual Geometry Group [25], used 30 segments of 10 s as the input and computed a probability for good outcome for every segment [24]. For the random forest model and the CNN, the probability for good outcome was defined as the average probability over 30 10-s segments. An overview of the models with respective inputs and outputs is shown in Fig. 1.

### Model Training and Validation

All models were trained and validated on data from Medisch Spectrum Twente and Rijnstate Hospital. We used fivefold cross-validation, in which 80% of the data were used for training and 20% of the data were used for internal validation. Models were trained for every hour from 4 to 72 h after cardiac arrest. Outcome of the models was the probability of good neurologic outcome, provided for every hour. Performance of the trained models was subsequently evaluated on the external independent datasets from the remaining centers. These external datasets have partially been used in previous work for validation, as well [24]. The training and evaluation of the models were performed in MATLAB, R (version 3.6.0, R Foundation for Statistical Computing, Vienna, Austria), and Python with Keras and Tensorflow by using a Nvidia graphics processing unit (GTX 980TI; Nvidia, Santa Clara, CA).

### Prognostic Performance

Receiver operating characteristic (ROC) analysis was performed. The mean ROC curve of all folds in the internal and external validation set, with corresponding 95% confidence intervals, was computed using threshold averaging. Overall model performance was quantified with the area under the ROC curve (AUC). The threshold values for prediction of good and poor outcome were chosen on the mean ROC curve of the training set for every hour. The threshold values corresponded with the highest sensitivity at > 90% and > 99% specificity for good and poor outcome prediction, respectively. In clinical practice, the prediction of poor outcome is only valuable when the specificity is almost perfect, as incorrectly predicting poor outcome could result in the withdrawal of life-sustaining treatment. For the prediction of good outcome, a slightly lower specificity can be used to identify, with high probability, patients with a large likelihood of good recovery.

The sensitivity and false positive rate (FPR) at these thresholds with their corresponding 95% confidence intervals were calculated in the internal validation and external test sets. Between-model differences in sensitivity for the reliable prediction of good and poor outcome in the test sets were tested by using McNemar’s test. A *p* value < 0.05 was assumed to reflect statistical significance. All tests were performed in MATLAB.

### Robustness to Artifacts

Robustness to artifacts was assessed by using data from Medisch Spectrum Twente and Rijnstate Hospital. The robustness of the models was analyzed by comparing the AUCs of the clean EEG epochs with the AUCs of epochs that were created with the artifact rejection algorithm turned off, epochs containing artificially added white noise, or epochs with random flat channels. Gaussian white noise was added to clean epochs to create epochs with a signal-to-noise ratio ranging from 7.5 to 9.5. Up to three EEG channels of clean epochs were randomly replaced with zeroes to create flat channels. All analyses and tests were performed in MATLAB.

## Results

### Patients

Out of the 929 patients included in this study, 58 patients were excluded from analyses because of loss to follow-up (*n* = 50) or by the artifact rejection algorithm (*n* = 8). Of the remaining 871 patients, 393 (45%) had good neurological outcome. Patient characteristics are presented in Table 1. Of the 32,391 5-min EEG epochs available for all patients within 4–72 h after cardiac arrest, 5430 (17%) were rejected because of artifacts.

**Table 1:** Patient characteristics and medication use in patients with good and poor outcomes

Characteristics	Good Outcome (*n* = 393)	Poor Outcome (*n* = 478)
Female sex, *n* (%)	80 (20)	129 (27)
Age, mean ± SD (year)	60 ± 12	65 ± 14
Out-of-hospital cardiac arrest, *n* (%)	367 (93)	430 (90)
Shockable rhythm, *n* (%)	359 (91)	267 (56)
Primary cardiac cause, *n* (%)	353 (90)	326 (68)
Targeted temperature management, *n* (%)	370 (94)	426 (89)
Treated with propofol, *n* (%)	334 (85)	385 (81)
Max propofol rate, mean ± SD (mg/kg/h)	3.2 ± 1.2	2.8 ± 1.1
Treated with midazolam, *n* (%)	108 (27)	121 (25)
Max midazolam rate, mean ± SD (µg/kg/h)	116 ± 70	126 ± 91
Treated with fentanyl, *n* (%)	154 (39)	204 (43)
Max fentanyl rate, mean ± SD (µg/kg/h)	1.6 ± 0.8	1.5 ± 0.8
Treated with remifentanil, *n* (%)	21 (5)	33 (7)
Max remifentanil rate, mean ± SD (µg/kg/h)	7.2 ± 4.4	4.4 ± 3.1
Treated with morphine, *n* (%)	185 (47)	175 (37)
Max morphine rate, mean ± SD (µg/kg/h)	26 ± 11	29 ± 17
Treated with sevoflurane, *n* (%)	21 (5)	30 (6)
End-tidal volume %, mean ± SD	1.4 ± 0.3	1.3 ± 0.3
Somatosensory evoked potential performed, *n* (%)	42 (11)	268 (56)
N20 bilaterally absent, *n* (%)	0 (0)	121 (25)

SD, standard deviation

### Model Training

**Fig. 2:**
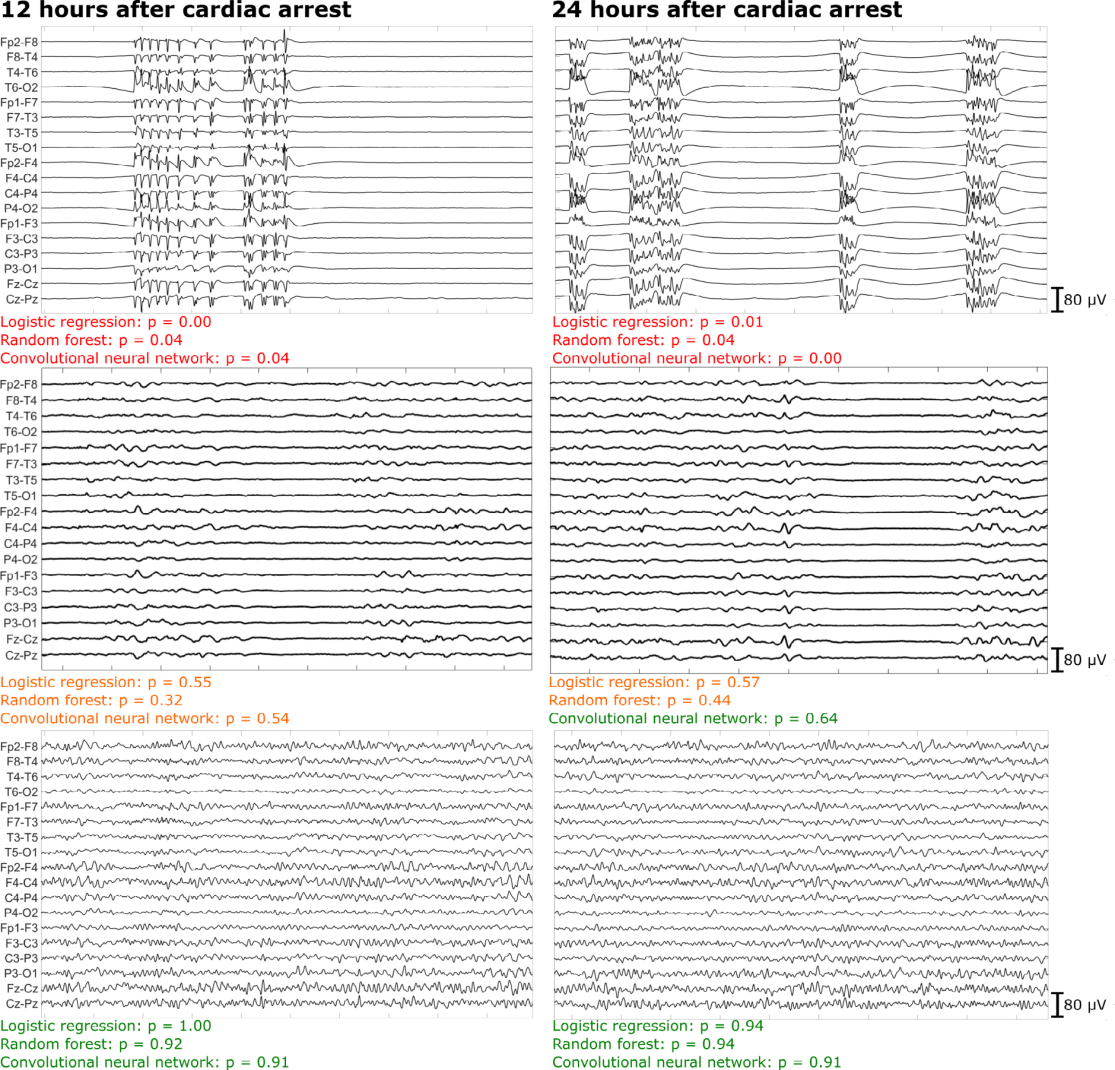
Examples of 10-s electroencephalogram (EEG) segments of three different patients at 12 and 24 h after cardiac arrest. The probability of good outcome predicted by the logistic regression model, random forest model, and convolutional neural network are shown below each panel. The colors denote the prediction of good (green), uncertain (orange), or poor (red) outcome of all models. Top: EEG segments of a patient with synchronous patterns at suppressed background, with very low probabilities of good outcome. This patient, indeed, had a poor neurologic outcome (Cerebral Performance Category [CPC] = 5). Middle: EEG segments of a patient with a discontinuous background pattern. At 12 h after cardiac arrest, all three models predicted an uncertain outcome. At 24 h after cardiac arrest, the logistic regression and random forest model still predicted an uncertain outcome, wheras the convolutional neural network correctly predicted a good outcome. This patient had a good neurologic outcome (CPC = 2). Bottom: EEG segments of a patient with early return to a continuous background pattern, with high probabilities of good outcome. This patient had a good neurologic outcome (CPC = 1)

The training dataset consisted out of 663 patients from the Medisch Spectrum Twente and the Rijnstate Hospital. EEG epochs of 290 and 469 patients were available at 12 and 24 h after cardiac arrest, respectively. Typical examples of EEG epochs and model outputs (predicted probabilities for good outcome) are shown in Fig. 2.

### Internal Validation

**Fig. 3:**
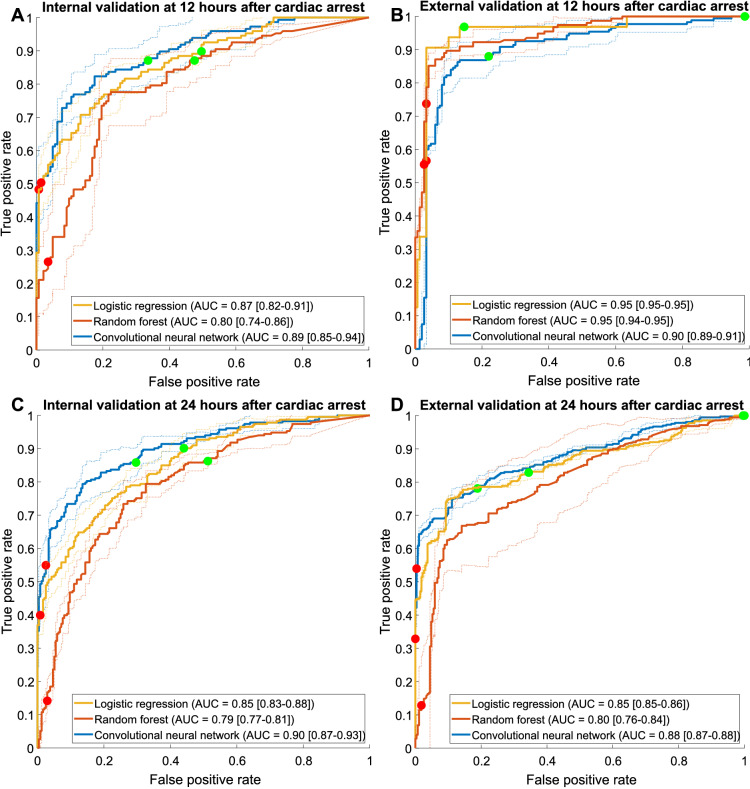
Average receiver operating characteristic (ROC) curves of the logistic regression model (yellow), random forest model (red), and convolutional neural network (blue). For the internal validation, ROC curves with corresponding 95% confidence interval (CI) are shown for all models at 12 (**a**) and 24 (**c**) hours after cardiac arrest. For the external test, ROC curves with corresponding 95% CIs are shown for all models at 12 (**b**) and 24 (**d**) hours after cardiac arrest. The solid red and green circles indicate the chosen thresholds in the training set for the prediction of poor and good outcomes, respectively. AUC = area under the curve

The ROC curves for the internal (cross-)validation of the models at 12 and 24 h after cardiac arrest are shown in Fig. 3. Overall model performance was the best for the CNN at 12 and 24 h after cardiac arrest (AUC of 0.89 at 12 h and AUC of 0.90 at 24 h). Sensitivities for reliable prediction of good and poor outcome with the respective FPRs are displayed in Table 2. At both 12 and 24 h after cardiac arrest, the CNN had higher sensitivity for reliable prediction of good outcome (67% at 13% FPR at 12 h, and 71% at 14% FPR at 24 h) than that of the logistic regression (sensitivity 51% at 9% FPR at 12 h, *p* = 0.06, and sensitivity 56% at 10% FPR at 24 h, *p* < 0.05) and random forest model (sensitivity 51% at 12% FPR at 12 h, *p* < 0.05, and sensitivity 48% at 14% FPR at 24 h, *p* < 0.05).

**Table 2:** Predictive values of the prediction algorithms, including 95% CIs, for the prediction of good and poor outcome at 12 and 24 h after cardiac arrest for the internal and external validation tests

Parameter	Internal validation			External validation		
	Logistic regression	Random forest	Convolutional neural network	Logistic regression	Random forest	Convolutional neural network
*Prediction of good outcome*						
Predictive threshold for > 90% specificity at 12 h	0.79	0.91	0.62	0.79	0.91	0.62
Sensitivity at 12 h in % (CI)	51 (32 to 70)	51 (10 to 92)	67 (34 to 100)	83 (83 to 83)	1 (0 to 4)	78 (52 to 100)
FPR at 12 h in % (CI)	9 (0 to 19)	12 (0 to 29)	13 (0 to 29)	3 (3 to 3)	0 (0 to 0)	12 (0 to 24)
Predictive threshold for > 90% specificity at 24 h	0.71	0.94	0.62	0.71	0.94	0.62
Sensitivity at 24 h in % (CI)	56 (39 to 73)	48 (20 to 75)	71 (59 to 83)	66 (55 to 76)	0 (0 to 0)	81 (72 to 90)
FPR at 24 h in % (CI)	10 (0 to 21)	14 (8 to 20)	14 (3 to 25)	17 (12 to 22)	0 (0 to 0)	22 (14 to 30)
*Prediction of poor outcome*						
Predictive threshold for > 99% specificity at 12 h	0.02	0.06	0.16	0.02	0.06	0.16
Sensitivity at 12 h in % (CI)	51 (30 to 72)	28 (0 to 63)	49 (18 to 81)	75 (75 to 75)	56 (31 to 81)	57 (43 to 71)
FPR at 12 h in % (CI)	1 (0 to 5)	4 (0 to 16)	1 (0 to 4)	3 (3 to 3)	3 (3 to 3)	3 (3 to 3)
Predictive threshold for > 99% specificity at 24 h	0.10	0.03	0.17	0.10	0.03	0.17
Sensitivity at 24 h in % (CI)	40 (24 to 55)	15 (5 to 24)	55 (34 to 76)	33 (33 to 33)	13 (0 to 50)	54 (44 to 64)
FPR at 24 h in % (CI)	1 (0 to 4)	3 (0 to 9)	2 (0 to 9)	0 (0 to 0)	0 (0 to 2)	0 (0 to 2)

CI, Confidence interval, FPR, false positive rate

**Fig. 4:**
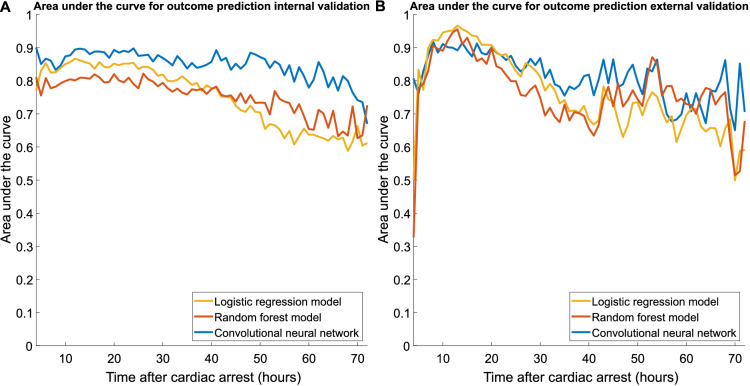
Area under the curve for the outcome prediction over a 4–72 h time period after cardiac arrest on the internal validation set (**a**) and the external test set (**b**)

Prediction of poor outcome at 12 h was best for the logistic regression model (sensitivity 51% at 1% FPR) and the CNN (sensitivity 49% at 1% FPR). Both models had significantly higher sensitivity than that of the random forest model (sensitivity 28% at 4% FPR, *p* < 0.05). For the prediction of poor outcome at 24 h, the CNN had significantly higher sensitivity (55% at 2% FPR) than that of the logistic regression (sensitivity 40% at 1% FPR, *p* < 0.05) and random forest model (sensitivity 15% at 3% FPR, *p* < 0.05). The CNN also showed the best overall performance in terms of AUC over the entire 4–72-h time period, as shown in Fig. 4.

### External Validation

The external dataset consisted of 208 patients from St. Antonius Hospital, University Medical Center Groningen, and VieCuri Medical Center. EEG epochs of 62 and 124 patients, respectively, were available at 12 h and 24 h after cardiac arrest.

The AUCs of the logistic regression model and random forest model were the same (0.95) and higher than the CNN (0.90) at 12 h after cardiac arrest (Fig. 3). At 24 h, the CNN showed the best performance (AUC 0.88). For reliable prediction of good outcome at 12 h after cardiac arrest, the logistic regression model (sensitivity 83% at 3% FPR) and CNN (sensitivity 78% at 12% FPR) performed significantly better than the random forest model (sensitivity 1% at 0% FPR, *p* < 0.05) (Table 2). At 24 h, the CNN (sensitivity 81% at 22% FPR) performed better than the logistic regression model (sensitivity 66% at 17% FPR, *p* = 0.11) and significantly better than the random forest model (sensitivity: 0% at 0% FPR, *p* < 0.05).

For the prediction of poor outcome at 12 h, the logistic regression model showed a significantly higher sensitivity (75% at 3% FPR) in comparison with the CNN (sensitivity 57% at 3% FPR, *p* < 0.05) and random forest model (sensitivity 56% at 3% FPR, *p* < 0.05). For the prediction of poor outcome at 24 h, the CNN obtained a significantly higher sensitivity (54% at 0% FPR) in comparison with the logistic regression (sensitivity 33% at 0% FPR, *p* < 0.05) and random forest model (sensitivity 13% at 0% FPR, *p* < 0.05). The CNN also showed the best overall performance in terms of AUC for up to 2 days after cardiac arrest, as shown in Fig. 4.

### Robustness to Artifacts

**Fig. 5:**
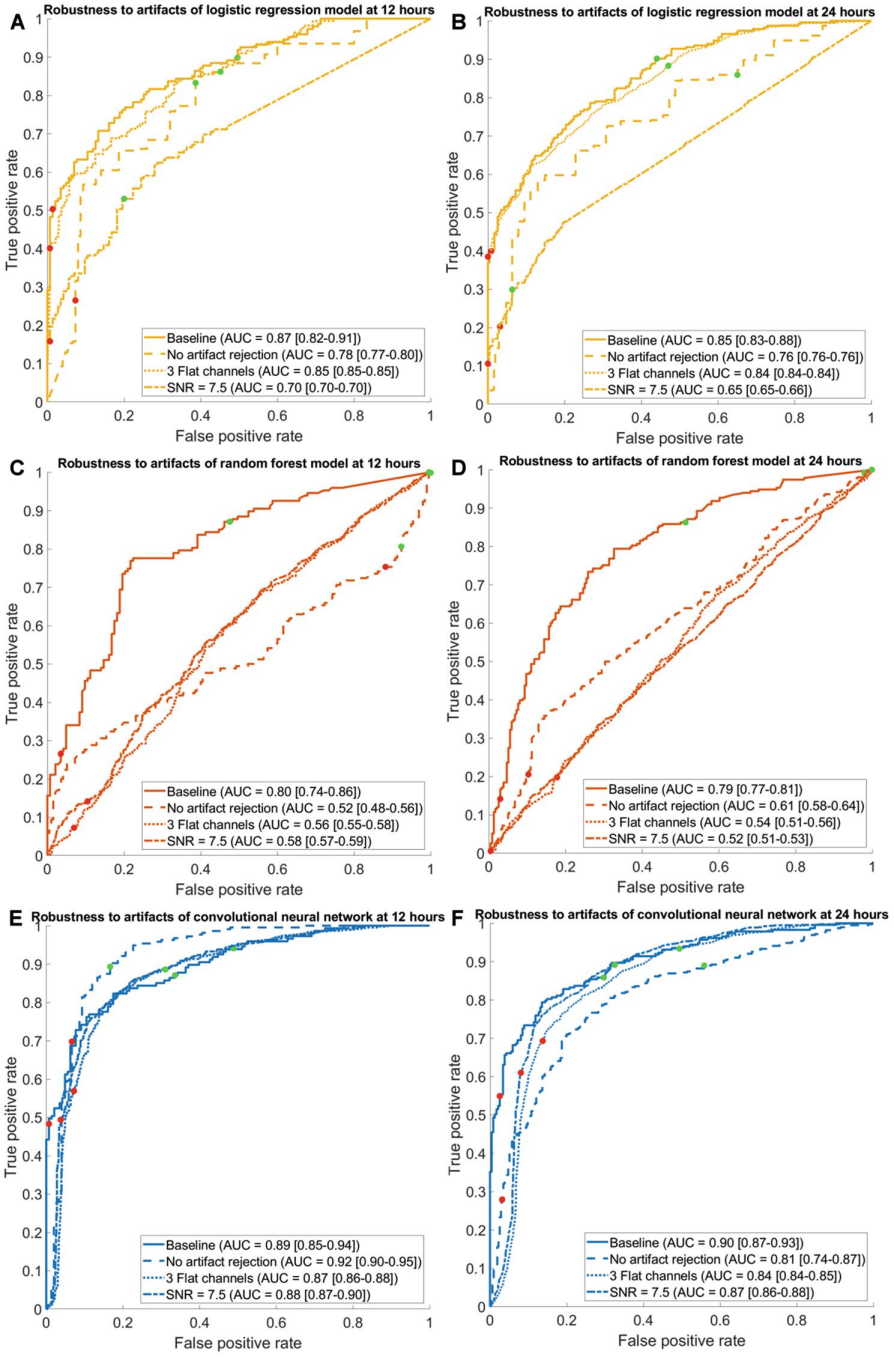
Average receiver operating characteristic (ROC) curves for the logistic regression model at 12 h (**a**) and 24 h (**b**) after cardiac arrest, the random forest model at 12 h (**c**) and 24 h (**d**) after cardiac arrest, and convolutional neural network at 12 h (**e**) and 24 h (**f**) after cardiac arrest. For every model, the ROC curves are shown for a baseline electroencephalogram (EEG) (solid line), EEGs without artifact rejection (dashed line), EEGs with flat channels (dotted line), and EEGs with additional Gaussian white noise (dash-dotted line). The convolutional neural network showed the best robustness to artifacts. AUC = area under the curve

The CNN showed the best results in terms of robustness to artifacts. The AUC of the CNN decreased the least compared with the other two models at 12 and 24 h after cardiac arrest when artifact rejection was not enabled, in EEGs with randomly flattened channels, and in EEGs with additional Gaussian white noise. The ROC curves and AUCs of all models during the artifact robustness testing are shown in Fig. 5.

## Discussion

We compared the performance of three EEG-based, computer-assisted prediction models for good and poor neurological outcome of comatose patients after cardiac arrest. A deep learning model based on a trained CNN performed better overall than logistic regression and random forest models in AUC and in sensitivity for prediction of good or poor outcome. Only in the first 12 h after cardiac arrest were the performance of the logistic regression and deep learning models comparable with each other for prediction of poor outcome. At an FPR < 1%, the CNN could predict poor outcome with a sensitivity of 44–64% up to 2 days after cardiac arrest. These predictive values for poor outcome are higher than reported values for visual EEG examination (sensitivity 47%) [6, 12, 18] or SSEP (sensitivity 28%) [4]. An important strength of a CNN is that it may also use EEG features that cannot be observed by a human reviewer, probably resulting in an optimal utilization of the integral richness of the EEG signal [25–27].

The performance of the CNN and logistic regression model are in line with our previous work [22, 24]. The logistic regression model performed particularly well during the first hours after cardiac arrest. This is because the regression model features were solely based on established visual EEG observations in patients with a poor outcome [22]. A prediction model only based on EEG continuity features will have strong performance early after cardiac arrest but will lose performance over time, when EEG continuity becomes less discriminative. The random forest model performed worse in this study than in our previous work [20, 21]. The models we used in 2017 were trained on a smaller dataset and were probably overestimated [20, 21].

Of the three models analyzed, the CNN was the most robust to artifacts. The removal of the artifact rejection algorithm, the addition of Gaussian white noise, or the randomized flattening of EEG channels had only small effects on its predictive performance. In CNNs, convolutions are applied over multiple channels and samples of the EEG signal, thereby incorporating both spatial and temporal information in feature extraction [24, 27]. This allows the CNN to overlook representations of the input that are nondiscriminative, such as artifacts, and instead “remain focused” on truly discriminative features in the input data [26]. In the logistic regression model and random forest model, the feature calculations are averaged over all EEG channels. The presence of an artifact in one or multiple channels will skew the average computed value of these features, which negatively affects prediction performance of these models.

We did not study what caused the differences in performance of the three models. In general, this is not trivial (and may differ per EEG pattern), as the logistic regression and random forest models are based on explicit features, whereas the deep learning model is not. Explainable deep learning may further provide information about the features used in the CNN [29]. However, such analysis was beyond the scope of our current analysis.

The use of sedative medication and mild hypothermia during targeted temperature management could have influenced our results [17]. However, we observed the opposite effect in previous work, in which higher doses of sedative medication were associated with better outcomes [22, 30]. We assume that patients with less severe brain injury are more likely to have a continuous EEG and have more arousals, often requiring more sedative medication. Recent data from a large observational study on hypothermia versus normothermia after out-of-hospital cardiac arrest also showed no difference in mortality or poor neurological outcome between the two groups [31].

A possible limitation of our analysis is that we used only one 5-min EEG epoch per hour. Predictive performance could be improved by using complete continuous recordings rather than 5-min epochs for every hour. The temporal evolution of the EEG during the ICU admission can provide additional information [32]. Other deep learning networks are optimized toward continuous temporal data [33, 34] and may further increase predictive values.

The prediction of neurological outcome can possibly be improved by combining patient data. Multimodal prediction models [35, 36], combining neurological examination, SSEP, and cerebral magnetic resonance imaging with the EEG may be tested in future studies.

## Conclusions

In this comparative study, a deep learning model based on a CNN was the best computer-based model for EEG-based outcome prediction of neurological outcome after cardiac arrest in predictive performance and robustness to artifacts. Its performance was better than logistic regression and random forest models and better than that reported for human visual EEG analysis. Prospective studies on effects of the implementation of computer-assisted EEG analysis on ICUs are needed.
